# Barriers and enablers to medicine-taking behaviours in chronic obstructive pulmonary disease: a qualitative interview study

**DOI:** 10.1007/s11096-025-01872-9

**Published:** 2025-02-05

**Authors:** Torbjørn Nygård, David Wright, Reidun L. S. Kjome, Hamde Nazar, Bernt Aarli, Aase Raddum

**Affiliations:** 1 Centre for Pharmacy, Department of Clinical Science, University of Bergen, P.O. Box 7804, 5020 Bergen, Norway; 2 School of Healthcare, University of Leicester, Leicester, UK; 3 Centre for Pharmacy, Department of Global Public Health and Primary Care, University of Bergen, Bergen, Norway; 4 Newcastle NIHR Patient Safety Research Collaboration, School of Pharmacy, Newcastle University, Newcastle Upon Tyne, UK; 5 Department of Clinical Science, University of Bergen, Bergen, Norway

**Keywords:** Chronic obstructive pulmonary disease, Health services research, Pharmaceutical services, Theoretical Domains Framework, Qualitative research

## Abstract

**Background:**

Chronic Obstructive Pulmonary Disease (COPD) is associated with low health-related quality of life and high costs to healthcare systems, particularly due to hospital admissions and exacerbations. Medicines, inhalers especially, reduce the risk of hospitalisations and exacerbations, but factors influencing medicine-taking behaviours are not fully understood.

**Aim:**

To explore experiences of people with COPD related to medicines, and followingly identify and characterise any barriers and enablers related to medicine-taking behaviours using the Theoretical Domains Framework (TDF).

**Method:**

Semi-structured qualitative interviews were conducted and included ten people with COPD who had previously been admitted to hospital. Systematic text condensation was used inductively in the primary analysis of the interviews. In the secondary analysis, meaning units from the primary analysis were mapped to the TDF and summarised as barriers and enablers.

**Results:**

Five major themes were developed in the primary analysis: (1) health literacy and information needs, (2) patient autonomy, (3) lack of access to medicines, (4) lack of effect from medicines, and (5) experiences of medicines-related issues. In the secondary analysis, thirteen barriers and nine enablers were mapped to nine out of the fourteen domains of the TDF.

**Conclusion:**

People with COPD experience challenges related to medicines which need to be addressed by researchers and healthcare providers. The identified barriers and enablers mapped to the TDF can guide and inform future design of interventions and health care services.

**Supplementary Information:**

The online version contains supplementary material available at 10.1007/s11096-025-01872-9.

## Impact statements

- People with COPD have unmet needs regarding provided care and their medicines which future interventions need to consider and account for in intervention design processes
- Mapping medicine-taking barriers and enablers to the theoretical domains framework provides future researchers and healthcare providers to develop and implement interventions underpinned by theory

## Introduction

Chronic obstructive pulmonary disease (COPD) is the fourth most common cause of death worldwide [1]. People with COPD suffer from respiratory symptoms with varying severity and are often also burdened with comorbidities such as cardiac diseases, depression, and anxiety. Generally, people with COPD have low health-related quality of life [2]. COPD is also associated with a high economic burden, in which exacerbations and hospital admissions are the main contributing factors [3]. Solutions to prevent hospital admissions are warranted.

COPD is a progressive and irreversible disease. Medicines, however, can alleviate symptoms and also reduce the risk of exacerbations, the number of hospital admissions, and the all-cause mortality [4, 5]. Polypharmacy—the use of several medicines simultaneously—is common in older adults with COPD and is associated with an increased risk of experiencing adverse drug reactions [6, 7]. Providing medicines support through multimodal interventions may reduce hospitalisations in COPD [8].

A systematic review from 2018 by Gardener et al. identified thirteen areas related to general support needs of people with COPD [9]. These areas included physical, psychological, and social needs, but information about medicines-related needs was limited. Other studies have found interventions which effectively improve outcomes such as adherence, disease control, health-related costs, and number of hospital admissions in people with COPD [10, 11]. However, interventions usually focus on single elements of care and are not underpinned by theory [12, 13].

The UK Medical Research Council’s guidance for developing and evaluating complex interventions recommends the use of theory to design interventions [14]. The theoretical domains framework (TDF) can be used to characterise barriers and enablers related to medicine-taking behaviours [15]. Furthermore, these barriers and enablers can be used to inform the development of interventions, using evidence-based approaches, for future researchers and healthcare providers [16, 17].

### Aim

To explore experiences of people with COPD related to medicines, and followingly identify and characterise any barriers and enablers related to medicine-taking behaviours using the TDF.

### Ethics approval

The Regional Committees for Medical and Health Research Ethics in Western Norway exempted this study from ethical approval (ref. 603326, 27.03.2023) because the study was considered as health services research.

## Method

### Study design

Semi-structured interviews were conducted and included people with COPD who had previously been admitted to hospital. The Consolidated Criteria for Reporting Qualitative Studies (COREQ) checklist was used to inform the reporting of this study (see Supplemental Material).

### Patient and public involvement

A user representative, SK, with lived experience of having COPD was included in this project. The user representative assisted in developing the interview guide, revising the interview guide, and pre-piloting the interview.

### Setting

This study was conducted in Western Norway. The pulmonary ward at Haukeland University hospital assisted with recruiting informants for this study. Interviews were conducted in the participants’ homes in Bergen and neighbouring municipalities.

### Recruitment

Participants were recruited by convenience sampling. Potential participants were eligible for inclusion if they had a diagnosis of COPD and if they had been hospitalised for COPD within the last year. Some of the participants were recruited from a previous questionnaire study in which hospitalised patients with COPD were included [18]. The respondents of the questionnaire had the opportunity to consent to be contacted later for interviews. The patients who had consented were contacted by telephone or e-mail directly. The other participants of this study were recruited from the hospital pulmonary ward, in which initial interest and consent to be contacted was acquired by a ward nurse or physician (BA). The potential participants who were interested were contacted one to two weeks after discharge from hospital to provide some time for recovery. Final written consent was obtained during the interviews. Carers or family members were invited to join the interviews as well according to the preferences of the patient.

### Data collection

Semi-structured interviews were conducted from January 2023 to February 2024. An interview guide (see Supplemental Material) was developed based on previous literature [12, 19–22]. The interview guide was piloted in the first two interviews, after revision and pre-piloting with assistance from the user representative. Minor changes were made to the interview guide after the pilot interviews to explore the post-discharge period and use of medicines in more detail. The main topics covered in the interview guide were “background information”, “hospital discharge and transition process”, “follow-up”, and “medicines”. All interviews were audio recorded with consent from each participant. Minor field notes were made during the interviews. The transcripts from the pilot interviews were sent back to the participants to check for comments and corrections. The pilot interviews were included in the final analysis. The sample size was estimated at ten interviews based on previous studies [23]. The sampling strategy was to reach sufficient quality and amount of information, quantified as two successive interviews which did not provide any changes to existing themes as per the definition of data generation saturation [24, 25].

### Analysis

The interviews were manually transcribed by TN. Transcription and analysis was done using MAXQDA 2024 (VERBI software). The verbatim transcripts were inductively coded and analysed using systematic text condensation [26]. The systematic text condensation was undertaken in four steps: (1) familiarisation with the data through reading the transcripts and identifying preliminary themes; (2) identifying meaning units (i.e., codes), organising meaning units based on preliminary themes, and sorting into code groups; (3) decontextualization of meaning units within code groups into condensates (i.e., summaries from participants’ point of view); (4) synthesising descriptive stories from condensates through reconceptualisation. Each step was undertaken by at least three members from the research team to facilitate a broader interpretation.

A secondary analysis was conducted by deductively coding the meaning units from the primary analysis into the TDF [15]. This was independently undertaken by TN and DW. Any discrepancies were reviewed by a third researcher, SS, with expertise in using the TDF. The findings were then summarised by TN into determinants and classified as barriers or enablers and double-checked by DW.

## Results

In total, 26 people with COPD consented to be contacted. Some of the patients were unavailable or were not well enough to participate. Five people were included from the previous questionnaire study and an additional five participants were needed to meet saturation. Three of the participants were female and seven were male. The age of the participants varied from being in their fifties to their eighties, and their COPD severity varied. In general, the younger participants had lower degree of disease severity. Each interview lasted between 45 and 60 min. Most participants had spouses, and some spouses participated in the interview. However, a few participants had no partner or lived by themselves. All participants lived at home, but some occasionally had temporary stays at intermediate care units or nursing homes. About half of the participants received home nursing or other home-based services, which included delivery of prescription medicines from pharmacy.

### Primary analysis

**Fig. 1:**
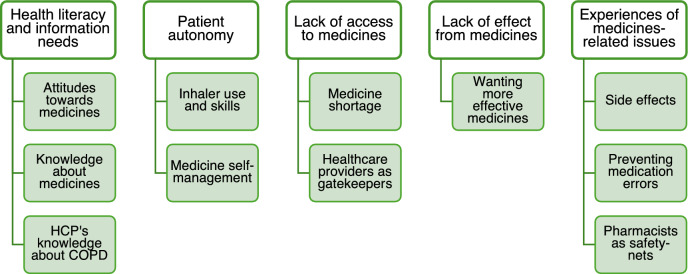
Coding tree of the primary analysis. HCP healthcare professional(s), *COPD* chronic obstructive pulmonary disease

In our primary analysis, we investigated descriptive content related to medicines and other information which could be relevant for pharmacists. We found five main themes in our primary analysis: (1) health literacy and information needs, (2) patient autonomy, (3) lack of access to medicines, (4) lack of effect from medicines, and (5) experiences of medicines-related issues (Fig. 1).

#### Health literacy and information needs

The participants of this study had varied attitudes towards medicines. Some of the informants were sceptical about medicines in general and disliked taking many medicines. Followingly, some were confused about the many different types and names of different inhalers. One participant stated that they used a pill organiser to simplify and limit the time and attention they spent on their medicines.

*I am not going to have any relation to my medicines, no relation at all, I am going to take my medicines because I must. And then I am finished with it. I am not going to take out pills one by one from one medicine box after another, because then it takes over your life. And I won’t have that*.

(*Participant 8*, *male*)

Confidence in knowledge varied from not seeking or needing additional information, to occasionally finding health information difficult to understand and being aware of their lack of knowledge about medicines. One participant described finding information difficult to retain during emotional and physical distress, such as during hospitalisations. Participants who attended pulmonary rehabilitation after hospitalisations highlighted that this provided them with useful knowledge about medicines.

A few of the participants believed that healthcare professionals generally lack knowledge about COPD treatment. Followingly, there was variability in the quality of information provided by healthcare professionals. Participants found it helpful when healthcare professionals initiated conversations about how to optimise inhaler treatment and experiences with side effects. On the other hand, they would sometimes feel dismissed when expressing concerns about side effects.

[…] *Because if you ask the doctor about [side effects], then they are very dismissive. They can reply with something like*: “*all tablets have side effects*” *and then they are done. And that doesn’t help much*.

(*Participant 10*, *male*)

#### Patient autonomy

Inhalers were used by all participants, but the self-perceived skill of correct usage varied. Experiences of practical issues with inhalers were common. Participants reported that they had received inhaler training from their general practitioner, in the pharmacy, or at pulmonary rehabilitation. When inhaler training was provided it was believed to be helpful. Still, a few participants stated that they had never received inhaler training, or they had no recollection of receiving prior inhaler training. In some cases, inhaler type was adapted after assessment from healthcare professionals because insufficient inhalation technique was detected. One participant emphasised that they found the inhaler training more useful when they could use demo-inhalers rather than their own device, as this allowed for repetition.

[*About inhaler training in pulmonary rehabilitation*] *Yeah, it was alright. I was fresh in the game related to the use of these kinds of medicines, so to get a more thorough introduction than what they say in the pharmacy, and also the doctor just prescribes it. No, so it’s a bit like, it’s very nice that they do it*.

(*Participant 8*, *male*)

Almost all participants managed their own medicines themselves, even those who received their medicines from their partners or home care nursing. Although patients self-managed their medicines, a few still needed help managing practical issues. One participant expressed frustration around constantly needing to ask for help with practical issues of medicines. This resulted in that patient no longer requesting help from home care nurses regarding these issues. If the participants had spouses or close relatives, then the relatives often provided help with medicines, such as getting medicines from the pharmacy. However, one participant highlighted that they did not want relatives to be too closely involved with their medicines, as this would put burden onto the relatives.

[…] *and for that matter [decongestant medicine], but it has a tendency to be forgotten, and I become tired of nagging about it*. […] *For them to help me with it, because it is not easy for me to do it alone*. […] *And I am tired of nagging so much, that’s what I’m thinking. Thus, things will unfold as they do, things could have well enough been avoided, if [home care nursing] would have been better at that part*.

(*Participant 6*, *female*)

#### Lack of access to medicines

Some of the patients had experienced that their medicines were unavailable to them. This was either because healthcare professionals were gatekeeping treatment or lack of access due to medicines shortage. Patients described episodes of healthcare providers denying them medicines: one patient described how they wanted an inhaler with greater effect, but the prescriber refused because the patient was not sick enough; other patients described not receiving treatment post-discharge because of healthcare providers’ negative attitude towards COPD or lack of knowledge about treating COPD; finally, one patient had also described that they had to be rehospitalised because the medicine they needed could not be provided in intermediate care.

[…] *I have had a short-term stay in nursing home*, *and [the nurse] screamed at me and waved her arms right up in my face, telling me*: “*you’ve recovered, you’re not supposed to have more* [*medicine*]!” […] “*There’s missing a tablet, the one from* [*the hospital*].” *And she exploded, “you’re not getting more medicine, you’ve recovered*!”

(*Participant 10*, *male*)

#### Lack of effect from medicines

A few of the participants had experienced no issues with their medicines, however, most had some complications related to their medicines. The biggest issue was that their medicines were perceived as not effective enough. Many of the patients wanted their medicines, especially their inhalers, to have a greater effect. However, some patients had found a balance between getting the effect they wanted from their medicines and taking too much. Many patients had good effect from short courses of oral corticosteroids during exacerbations, but some also believed that the full effect was only achieved when oral corticosteroids were combined with antibiotics. In general, the patients thought that their medicines helped to some degree, because it was noticeable if they did not take their medicines.

[…] [*inhalers*] *are not medicines which are developed for patients with COPD. And there is no medicine today*. *It’s only for asthma. But, of course, it does help. But it’s not as effective as other medicines, you could say*.

(*Participant 3*, *male*)

#### Experiences of medicines-related issues

Almost all the patients had experienced side effects as complications with their medicines. The patients had experienced dry mouth and tachycardia from their inhalers, nightmares from smoking cessation medicines, general discomfort and cardiovascular side effects from oral corticosteroids, and gastrointestinal side effects (i.e., nausea) from antibiotics. In many cases, the side effects outweighed the benefits of the medicines, resulting in quitting or changing their treatment. One participant took short breaks from certain medicines to avoid getting side effects. Participants had also experienced side effects from non-proprietary medicines, which caused negative attitudes towards non-proprietary medicines and a preference for proprietary branded medicines. A few participants feared getting side effects, especially from new medicines they had not previously used. One participant described how they were negatively influenced by reading about others’ experiences with medicines, which made them postpone starting their newly prescribed medicines.

*When I read the list of side effects, then I don’t want to start using them. Right, because I’ve been okay without. But of course, it would have probably been easier if I used the medicines, right. But I’m a bit afraid of getting side effects, right. In such bad shape from before, so I don’t want side effects on top of it all. And what I’ve read about the* [*medicines*] *now, is that there are some that get side effects from it*.

(*Participant 5*, *female*)

Some of the participants had received continuous assessment of their medicines while hospitalised, however, a few participants had experienced episodes of medicine errors while in hospital. A few participants had also been provided assessment and counselling regarding their medicines in the hospital-led pulmonary rehabilitation. A few patients described preventative measures they took at home, such as monitoring oxygen saturation for early detection of exacerbations or having rescue packs of oral corticosteroids and antibiotics at home.

Patients recognised the contribution pharmacists can make in terms of medicines, such as detecting interactions or providing information about medicines and side effects. Also, because actual use of inhalers can differ from prescriptions or recommendations from physicians, it was helpful when pharmacists could find solutions to this problem. Most of the information the participants received in pharmacies was usually focused on new medicines only.

[…] *No, I don’t think I received* [*information about medicines at the pharmacy*]. *No, I don’t think I did. But it was medicines I had used before*. […] *I have used* [*my medicines*] *for many years, right, the same type. But if I get new medicines, then they do go through them at the pharmacy*. […]

(*Participant 5*, *female*)

### Secondary analysis

Inductive codes from the primary analysis were sorted into the TDF and summarised into determinants. Nine out of the fourteen domains of the TDF were used to describe the codes. The determinants were separated into barriers and enablers within the domains by TN and checked by DW (Tables 1 and 2).

**Table 1:** Medicines-related barriers mapped to the Theoretical Domains Framework (TDF)

TDF domain	Barriers	Comments
1. Knowledge	Lack of information about medicines	Includes side effects. Alternative sources used due to lack of information from HCP
	Negative perceptions towards inhalers	Believes that inhalers are developed for asthma, not COPD
2. Skills	Practical issues with inhalers	Experience of practical issues with inhalers and lack of inhaler training
4. Beliefs about Capabilities	Reduced access to health services due to immobility	Unable to easily visit pharmacies and other healthcare institutions
6. Beliefs about Consequences	Concerns about side effects	Past experiences of side effects and other adverse reactions leading to concerns
	Lack of effect from medicines	Medicines, especially inhalers, not providing the expected effect
10. Memory, Attention and Decision Processes	Forgetting to pick up medicines	Forgetting medicines and/or vaccines when visiting pharmacies
	Confusion around the many types of inhalers	Use of multiple different inhaler types and changing between different inhalers
11. Environmental Context and Resources	Low availability of post-discharge follow-up	Lack of follow-up. Medicine shortages or healthcare providers reducing access to medicines
	Non-receptiveness for information	Not receptive for information during exacerbations and/or hospitalisations
12. Social influences	Burdening family with responsibility for medicines	Reduces involvement of relatives with medicine regime to reduce burden
13. Emotion	Fear of getting side effects	Does not start taking new medicines out of fear of getting side effects
	Frustration around taking medicines	Frustrated that they need to take medicines

*HCP* healthcare professional(s), *COPD* chronic obstructive pulmonary disease

**Table 2:** Medicines-related enablers mapped to the Theoretical Domains Framework (TDF)

TDF domain	Enablers	Comments
1. Knowledge	Information about medicines provided by healthcare professionals	Information about side effects or written information
	Strategies to prevent or treat side effects	Knowledge about which side effects could be expected and what could be done to prevent or treat them
2. Skills	Inhaler training and assessment	Training in pulmonary rehabilitation or pharmacy. Training with demo-inhalers. Being assessed by HCP
	Adapting medicines according to needs	Self-adapting or with help from HCP based on patient needs
4. Beliefs about Capabilities	Self-managing medicines	Capability of being in control of their medicines—independence/autonomy
	Self-monitoring disease to detect exacerbations	Monitoring clinical parameters (e.g., pulse oximetry)
5. Optimism	Wanting medicines with greater effect	Hopefulness about the existence of and development of more effective medicines
6. Beliefs about Consequences	Early antibiotics and oral corticosteroids prevent exacerbations	Belief that antibiotics and oral corticosteroids could prevent exacerbations/hospitalisations if taken early enough
11. Environmental Context and Resources	Access to medicines in pharmacy or at home	Increased access to medicines through rescue packs

*HCP* healthcare professional(s)

## Discussion

### Key findings

The research has identified that patients with COPD included in this study have unmet needs for information regarding both their health and medicines. Furthermore, some believed that healthcare providers lacked sufficient knowledge of COPD and its treatment. There was a perceived lack of willingness by healthcare professionals to provide information regarding side effects, which patients wanted to enable them to adapt or change their treatment.

The offer of training in inhaler technique and allowing patients to manage their own medicines were both seen as enabling better medicines use. With the right information and support, patients wanted autonomy to manage their medicines, monitor their disease and identify exacerbations early so they could prevent their condition from worsening.

Whilst medicines shortages are an obvious barrier to accessing medicines in a timely manner, some patients reported healthcare providers acting as gatekeepers (i.e., preventing treatment options) as an additional barrier.

### Strengths and weaknesses

The systematic review by Gardener et al. had similar findings as this study, especially in terms of the need for knowledge about COPD and how to manage symptoms and medicines [9]. Gardener et al. also reported on the emotional and social needs, which was not within the scope of this study. Compared to the study by Gardener et al., this study takes the analysis one step further by using the TDF. The translation of identified barriers and enablers to domains allows researchers to develop complex medicines-related interventions informed by theory.

The use of the TDF for interpreting the results allows for a theory-based understanding of which factors to focus on when developing healthcare interventions. The TDF is a validated framework commonly used in implementation science. Therefore, using the TDF ensures that recommendations for future health service research are informed by theory. Followingly, our use of the TDF can help pharmacists choose appropriate behaviour change techniques when targeting medicines-related behaviours.

The participants included in this study were patients with experience from hospitalisations due to COPD. Many of the patients were hospitalised several times with various experiences of care. This provided interviews with rich information about past experiences. Although the patients had varied experiences, main themes were developed early on and ten patients provided sufficient information to report findings. However, participants were only recruited from one hospital ward which limits the transferability to other contexts. Including patients from hospitals in other regions or countries would have increased transferability of this study.

Investigator triangulation was undertaken to improve credibility of this study. Multiple researchers were included in each step of the analysis, which facilitated breadth in perspectives and interpretations. Furthermore, we included a physician to bring non-pharmacist perspectives to the results. The secondary analysis also included several researchers, in which an expert in the TDF reviewed the initial coding to facilitate a rigorous analysis. Additionally, this study included an experienced user representative with COPD. The user representative helped with the development of the interview guide and pilot testing. However, the user representative was not included in the analysis, which could have improved the credibility of findings. Credibility could have been further improved by member checking for all participants.

Convenience sampling, although not ideal, was used to sample participants. The sampling method was chosen because a low recruitment rate was expected from the previous questionnaire study and due to time constraints [18]. Sampling methods such as purposive sampling should have been used. Also, alternative methods to assess sample size would have been preferred, such as using information power [27].

### Interpretation and further research

The findings from this study suggest that people with COPD have a need for information about their disease and their medicines. Lack of knowledge may be the reason behind scepticism and negative perceptions towards their medicines. Pulmonary rehabilitation was highlighted by the participants as a useful service to provide knowledge and skills about medicines. Pulmonary rehabilitation is recommended by GOLD, the American Thoracic Society, and the European Respiratory Society [2, 28] and is shown to improve health-related quality of life in people with COPD [29]. However, barriers related to pulmonary rehabilitation exist and there is a need to individualise such services for each patient [12, 30]. Thus, researchers and providers of pulmonary rehabilitation need to identify optimal strategies to provide medicines-related information for people with COPD and how to individualise the service for patients.

The participants in this study reported concerns regarding lack knowledge about COPD among healthcare professionals. Perhaps of greater concern was that the participants reported experiences of feeling stigmatised by healthcare providers, which may negatively impact provision of treatment and patient satisfaction with care [31]. When creating interventions to support people with COPD, training to prevent healthcare provider stigmatisation and patient self-stigmatisation should be included within it.

Results from this study, in line with new GOLD report recommendations [2], show that there is a need for inhaler training. Even though some of the participants had received inhaler training previously, the amount of training and confidence in use varied greatly between participants. In Norway, there is an inhalation technique assessment service in pharmacies [32] for people with asthma and COPD. However, some of the participants reported not receiving inhaler training and some did not visit pharmacies regularly. National information campaigns about such services aimed at both healthcare providers and patients should be undertaken to increase reach. Also, implementing the service in additional areas of the healthcare system may prove beneficial if patients do not visit pharmacies. Other countries adopting similar services should investigate which areas would provide the highest reach as patients with COPD may be home-dwelling.

Non-proprietary inhalers are provided in Norwegian community pharmacies as a fully reimbursable alternative to proprietary inhalers. Therefore, the patient’s choice of inhaler is determined at the point of supply in the pharmacy. Non-proprietary inhalers often need different inhalation techniques compared to proprietary inhalers, thus there may be discrepancies between training provided by nurses or physicians and in pharmacies. Internationally, patients may benefit from subsidised inhaler treatment to ensure continuity in provided inhaler device or that inhaler training is provided at the point of supply to ensure that the training provided matches the dispensed device.

Participants in this study valued having autonomy of treatment and being in control of their own medicines. Increased engagement and empowerment in patients are recommended by the World Health Organization through the “Patients for patient safety”-programme [33]. Interventions which focus on self-management aim to empower people managing chronic diseases. Furthermore, self-management interventions are associated with increased health-related quality of life and a lower risk of COPD-related hospitalisations [34]. Whilst healthcare providers should support people with COPD to enable them to self-manage their disease and medicines, effective approaches to achieve this are required.

Medicine shortages are becoming increasingly more common, which may affect the access to important medicines such as inhalers, antibiotics, or oral corticosteroids. With frequent rehospitalisation being a significant problem within this population, prioritising access to important medicines for people with COPD is warranted, especially in countries with lower resources [35]. Patients identified the value of holding rescue packs of medicines at home for use during early exacerbations, which may prove beneficial for people with COPD to prevent hospitalisation. This usually involves holding antibiotics and steroids at home to prevent exacerbations from progressing. However, it is important that a patient’s ability to self-medicate appropriately is ascertained prior to implementation [36].

## Conclusion

People with COPD experience challenges with their medicines which need to be addressed by healthcare providers and researchers. Rigorous research is needed to optimise healthcare provision in terms of access to and availability of healthcare services, continuity of care across care settings, and empowerment of patients. Also, actions to reduce stigma towards people with COPD are warranted. The use of the TDF enables future researchers and healthcare providers to identify effective behaviour change techniques to include in future interventions which may address issues reported in this study.

## Supplementary Information

Below is the link to the electronic supplementary material.

Supplementary file1 (PDF 408 kb)
